# What’s That in the Aorta? A Case of Asymptomatic Dislodged Ostial Right Coronary Artery Stent That Was Noted as an Echodense Material in the Aortic Valve on Transesophageal Echocardiogram

**DOI:** 10.7759/cureus.16120

**Published:** 2021-07-02

**Authors:** Anjali R Desai, Sravani Avula, John Rashid

**Affiliations:** 1 Internal Medicine, University of Illinois College of Medicine at Peoria, Peoria, USA; 2 Cardiology, OSF HealthCare/University of Illinois College of Medicine at Peoria, Peoria, USA; 3 Interventional Cardiology, University of Illinois College of Medicine at Peoria, Peoria, USA

**Keywords:** ostial stent, dislodged stent

## Abstract

A 58-year-old female with a history of coronary artery disease (CAD) with remote percutaneous intervention (PCI) to ostial right coronary artery (RCA) with a bare-metal stent represented with unstable angina. Left heart catheterization (LHC) showed 90% stenosis of the previously stented ostial RCA with a moderate disease in the circumflex and left anterior descending arteries (LAD). LHC had also demonstrated that the previously placed ostial RCA stent, 19 years ago, was dislodged with only 3-4 mm within RCA and the remainder 10-12 mm in the ascending aorta. The patient miraculously had remained largely asymptomatic of this dislodged RCA stent for many years. Subsequent transthoracic echo (TTE) showed moderate-severe mitral regurgitation (MR). Therefore, she was worked up for a possible single-vessel coronary artery bypass graft surgery (CABG) with mitral valve replacement/repair. However, on transesophageal echo (TEE), MR was noted to be moderate in severity. Also, an echodense material was noted on the right coronary cusp (RCC) of the aortic valve, which was deemed to be the dislodged RCA stent. As the MR was moderate, the patient underwent successful complex PCI of ostial RCA.

## Introduction

Coronary artery stent dislodgement is a rare but life-threatening complication of percutaneous intervention (PCI). It can cause embolic phenomenon, dissection, or even death [1]. Most commonly, these dislodged stents have had to be emergently retrieved intravascularly or surgically [2-4]. We present a rare case of a 19-year-old ostial right coronary artery (RCA) stent which was noted to be dislodged without symptoms for several years.

## Case presentation

**Figure 1:**
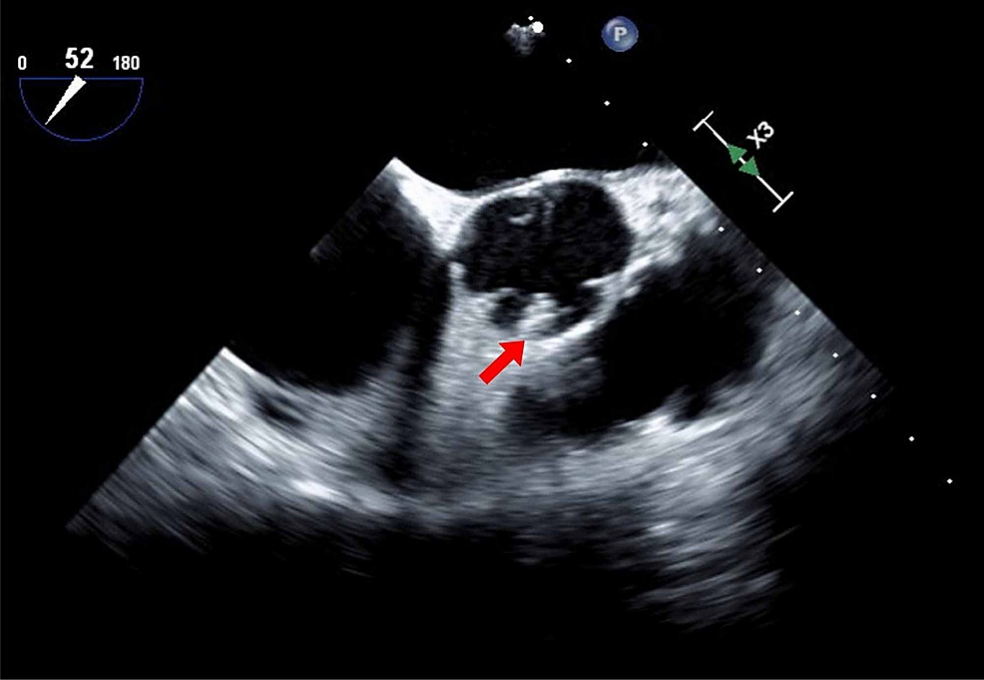
RCA ostial stent dislodged into the aorta (arrow) RCA: right coronary artery

A 58-year-old female with a remote history of coronary artery disease (CAD) with PCI to ostial RCA with bare-metal stent 19 years ago presented with chest pressure radiating into her shoulders concerning for unstable angina. EKG showed ST-segment depressions in the anterolateral leads. Nuclear medicine pharmacological stress test that was performed few days prior showed diffuse ST-segment depression but no myocardial perfusion defects. She was transferred to our tertiary care center for cardiac catheterization. Left heart catheterization (LHC) showed 90% stenosis of the previously stented ostial RCA. The LHC also demonstrated that the previously placed ostial RCA stent was dislodged with only 3-4 mm within RCA and the remainder 10-12 mm in the ascending aorta (Figure 1).

**Figure 2:**
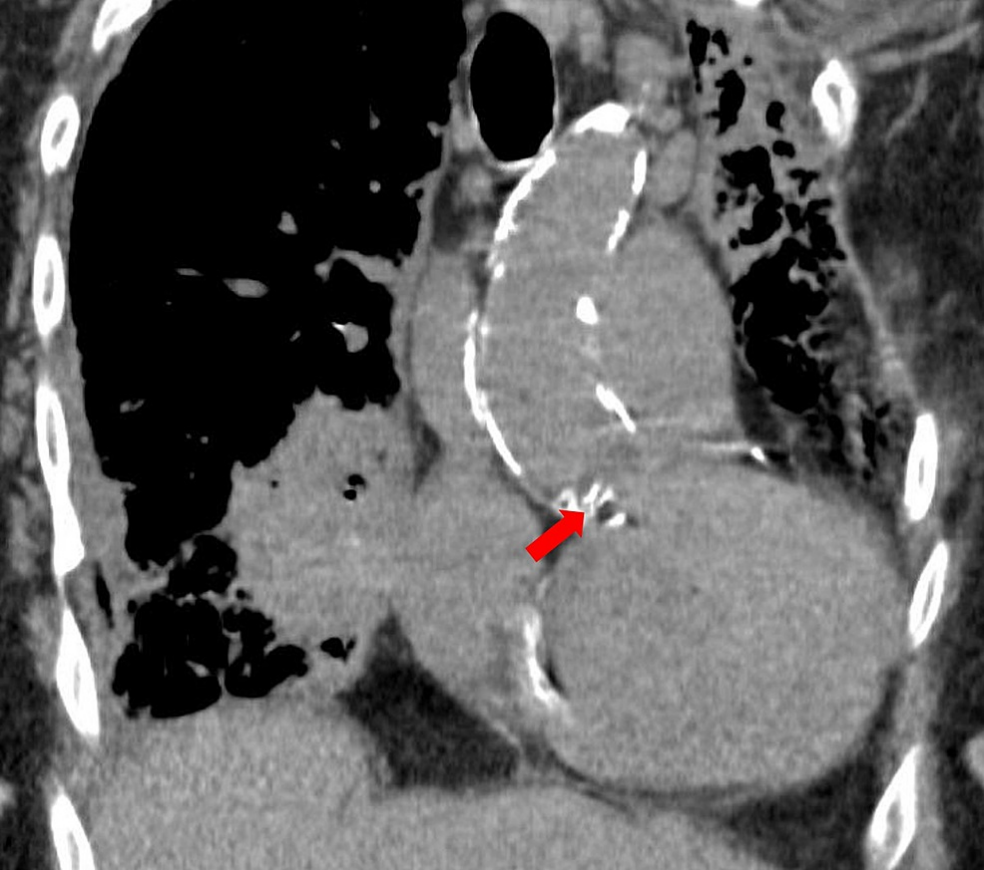
CT chest without contrast showing dislodged RCA stent RCA: right coronary artery

In addition, transthoracic echo (TTE) during this admission showed moderate to severe mitral regurgitation (MR), presumably due to RCA lesion. Thus, PCI was deferred at that time, in case the patient needed mitral valve replacement or repair plus single-vessel coronary artery bypass graft surgery (CABG). For workup, a transesophageal echocardiogram (TEE) was performed and showed only moderate MR. However, an echodense structure was noted in the right coronary cusp (RCC) of the aortic valve that was fairly immobile. Upon further investigation, this material was thought to be the dislodged RCA stent (Figure 2).

Finally, given that her MR intensity was only moderate, complex PCI of ostial RCA was performed. Extra effort was made to flare the old ostial stent to try to oppose the old stent as much as possible to the aortic cusp before reintervention to the ostial RCA with a new stent. The 90% stenosed ostial RCA was ballooned and stented with 3 x 12 mm XIENCE™ (Abbott, Chicago, USA) drug-eluting stent (DES) reducing 90% stenosis to 0% residual. The mid LAD had 70% eccentric calcific disease that was proven to be hemodynamically significant with an instantaneous wave-free ratio (IFR) of 0.62, which was reduced to 0% with 3 x 12 mm Xience DES.

## Discussion

Coronary artery stent dislodgement is a rare complication of PCI. They seem to occur due to inadequate stent deployment due to coronary artery angulation, severe coronary calcification, or direct stenting [1]. The prevalence of stent dislodgement has been reported to be high in the left main and LAD, and lower in left circumflex and RCA arteries [4]. Interestingly in this case, besides worsening of ostial RCA disease, this dislodged stent was largely asymptomatic for many years. We argue that the ostial location of the stent made it symptomatic, not its dislodgement. The stent most likely had remained in place for at least 13 years since initial deployment because LHC six years ago showed that the stent was intact and patent. Another interesting aspect of this case was the visualization of the dislodged stent in the short axis of the aortic valve on TEE as an echodense structure. Differential for such echodense structures in the aortic valve generally includes vegetations, thrombi, papillary fibroelastoma, myxomas, or Lambl’s excrescences [5,6] but based on the immobility and position in the RCC of the aortic valve, it was deemed to be the dislodged stent. 

## Conclusions

Coronary artery stent dislodgement is mostly fatal, requiring emergent retrieval. However, partially dislodged stents could rarely be asymptomatic as in this case.
